# Treatment of Stress Urinary Incontinence with Muscle Stem Cells and Stem Cell Components: Chances, Challenges and Future Prospects

**DOI:** 10.3390/ijms22083981

**Published:** 2021-04-12

**Authors:** Florian A. Schmid, J. Koudy Williams, Thomas M. Kessler, Arnulf Stenzl, Wilhelm K. Aicher, Karl-Erik Andersson, Daniel Eberli

**Affiliations:** 1 Department of Urology, University Hospital Zurich, University of Zurich, 8091 Zurich, Switzerland; florian.schmid@usz.ch; 2 Institute of Regenerative Medicine, Wake Forest University School of Medicine, Winston Salem, NC 27101, USA; kwilliam@wakehealth.edu (J.K.W.); karl-erik.andersson@med.lu.se (K.-E.A.); 3 Department of Neuro-Urology, Balgrist University Hospital, University of Zurich, 8008 Zurich, Switzerland; tkessler@gmx.ch; 4 Department of Urology, University Hospital Tubingen, University of Tubingen, 72076 Tubingen, Germany; urologie@med.uni-tuebingen.de (A.S.); aicher@uni-tuebingen.de (W.K.A.)

**Keywords:** stress urinary incontinence, treatment, regenerative medicine, muscle stem cells

## Abstract

Urinary incontinence (UI) is a major problem in health care and more than 400 million people worldwide suffer from involuntary loss of urine. With an increase in the aging population, UI is likely to become even more prominent over the next decades and the economic burden is substantial. Among the different subtypes of UI, stress urinary incontinence (SUI) is the most prevalent and focus of this review. The main underlying causes for SUI are pregnancy and childbirth, accidents with direct trauma to the pelvis or medical treatments that affect the pelvic floor, such as surgery or irradiation. Conservative approaches for the treatment of SUI are pelvic physiotherapy, behavioral and lifestyle changes, and the use of pessaries. Current surgical treatment options include slings, colposuspensions, bulking agents and artificial urinary sphincters. These treatments have limitations with effectiveness and bear the risk of long-term side effects. Furthermore, surgical options do not treat the underlying pathophysiological causes of SUI. Thus, there is an urgent need for alternative treatments, which are effective, minimally invasive and have only a limited risk for adverse effects. Regenerative medicine is an emerging field, focusing on the repair, replacement or regeneration of human tissues and organs using precursor cells and their components. This article critically reviews recent advances in the therapeutic strategies for the management of SUI and outlines future possibilities and challenges.

## 1. Introduction

The involuntary loss of urine remains a major medical issue with approximately 20% of people affected at some point during their lifetime [1,2] and a three-to-one ratio between women and men [3,4]. Patients suffering from urinary incontinence (UI) experience a severely reduced quality of life (QoL) [5,6] and there is an unmet medical need including continuously rising healthcare costs [7,8,9,10]. Among different types of UI, this article focuses on stress urinary incontinence (SUI), the most prevalent subtype [1,11]. The International Continence Society (ICS) defines SUI as “complaint of involuntary loss of urine on effort or physical exertion including sporting activities, or on sneezing or coughing” [12]. The etiology of this symptom is manifold but often caused by previous injury to the pelvic floor. Damage can occur during childbirth or surgical treatments, radiotherapy of tumors, as consequence of trauma to the pelvic floor or as an effect of aging [11,13]. Contributing risk factors include obesity, smoking, the number of pregnancies and vaginal deliveries, menopause, polypharmacy and the presence of lung diseases causing chronic cough [14]. Current treatment options enable the recovery of continence with various outcomes [15]. However, until today, a definite solution for the correction of the underlying etiology is lacking. Furthermore, the actual treatments only offer short-term relief, and the overall success of these therapies is mostly limited due to complications and their sequelae [16]. The search for new solutions and different treatments has been ongoing for decades, but without any great success. However, tissue engineering (TE) utilizing stem cells has emerged as a promising regenerative and minimal-invasive solution for this multifaceted disease [17].

## 2. Stress Urinary Incontinence

SUI is a major medical issue. Approximately 50% of the female population over 45 years is affected and around 20% of men after 70 years of age [3,7,18]. There are several triggers for urine leakage in SUI including coughing, laughing, sneezing, exercising and other movements that increase the intra-abdominal pressure on the bladder that provoke loss of urine in patients. Importantly for this type of UI, the leakage happens in the absence of detrusor contraction.

The QoL in patients with SUI can be severely reduced due to limited daily activities and the exposure to unpleasant sensation and odor caused by wet diapers, which often leads to recurrent urinary tract infections [19]. SUI further affects sexuality and the personal well-being, which provokes shame, depression, impaired productivity at work, reduced employment and a higher possibility to be admitted to a nursing home at higher age [6,11].

The worldwide healthcare expenditures for the treatment of this condition are constantly increasing. In the USA, it is estimated that the direct cost of caring for patients with UI adds up to around $20 billion dollars per year [9,20,21].

### 2.1. Pathophysiology

Continence and micturition are achieved by a complex interplay of anatomical structures such as the urethral sphincter, detrusor, bladder neck, urethral smooth muscle, nerves, vascular plexus and the surrounding tissue support [22]. There is an active and passive component contributing to intraurethral pressure. This pressure is mainly generated by the rhabdosphincter (external urethral sphincter, striated muscles, intentional) and the lissosphincter (internal urethral sphincter or “Hessian loop”, smooth muscles, unintentional)–both belonging to the active component [23,24]. Normally, the urethral pressure exceeds bladder pressure, resulting in continence. The proximal urethra and bladder are both within the pelvis and an increased intra-abdominal pressure is transmitted to both urethra and bladder equally. In case of an intact pelvic floor, this leaves the pressure differential unchanged and therefore results in urine remaining within the bladder (passive component). Normal voiding is initiated by the parasympathetic nervous system through the pelvic nerve. As a result, urethral pressure falls (relaxation of sphincter) and bladder pressure rises (detrusor contraction) [22]. During the filling phase, sympathetic nervous activity inhibits the detrusor and activates the smooth urethral muscles (unintentional), increasing the outlet resistance through hypogastric nerves and stimulation of alpha-adrenoreceptors [24]. In addition, efferent pudendal nerve activity increases the tonicity of the striated urethral sphincter (intentional). The sphincter complex prevents UI by providing both resting urethral tone via slow-twitch fibers and rapid reflexive contraction, if the abdominal pressure rises quickly. However, the external urethral sphincter (EUS) is, for the most part, responsible for preventing involuntary urine leakage. Therefore, any damage to the EUS, pelvic floor, nerves or surrounding tissue may lead to SUI.

A further important term is intrinsic sphincter deficiency (ISD), which tries to subsume muscular sphincter dysfunction. However, experts often disagree on the meaning and implications of ISD. Clinically, it is mostly defined as a combination of severe SUI due to low maximal urethral closure pressure (MUCP) and a low Valsalva leak point pressure (VLPP) [25,26,27]. ISD can occur with or without the presence of a hypermobile urethra [15]. Irrespective of pathophysiology, SUI is the result of high intra-abdominal pressure that exceeds the urethral and pelvic floor pressure in the setting of a sudden increase of intra-abdominal forces [28,29].

### 2.2. Etiology and Diagnostics

The etiology of SUI differs between men and women. In women, the cause of poor urethral sphincter function is neither fully known, nor entirely understood. Nevertheless, several important risk factors have been identified such as body mass index (BMI) and advancing age. With increasing age, the loss of muscle fibers—turning into scar tissue—goes along with the denervation of relevant structures of the pelvic floor. The number of pregnancies with subsequent vaginal deliveries relevantly influences this development. Therefore, nulliparous women or those having delivered by Cesarean section experience a much lower risk for SUI. Further, hormonal changes during menopause, smoking, chronic obstipation, cognitive impairment, irradiation or post-surgical complications all potentially contribute to the development of SUI [14]. In addition, anatomical alteration, such as a hypermobile urethra or pelvic organ prolapse, can also predispose for SUI. Interestingly, symptoms usually worsen before menstruation. These facts imply that the estrogen and progesterone level may have a relevant impact on urethral and sphincter function. As pathophysiological explanation, lowered estrogen levels lead to reduced muscular pressure around the urethra and therefore increasing the chances of involuntary leakage [30,31]. In men, the main causes for SUI are the adverse effects of radical prostatectomy. Despite improved surgical techniques, persistent SUI rates have shown to vary between 10 and 30% [32].

According to the classification of Stamey, there are three grades of clinical SUI: Urine leakage while coughing, sneezing or laughing (grade 1), while walking, climbing the stairs or standing up (grade 2) or while lying horizontally (grade 3) [33]. The severity of SUI can be easily quantified with bladder diaries, pad-tests and questionnaires. In complex cases with suspicion of mixed UI or in order to clearly differentiate between the subtypes of UI, a urodynamic investigation should be performed [34]. With urodynamics, the urologist wants to rule out potential concomitant diseases from the urinary tract, which would hamper the operative result [35]. Before planning a specific therapy, a sonographic examination of the whole urinary tract (including uroflowmetry with post void residual volume testing) and the exclusion of a lower urinary tract infection is needed [36].

### 2.3. Treatment

Various treatment options are available for patients suffering from SUI. Most importantly, the choice of a specific therapeutic pathway depends on the clinical severity and objective psychological burden and must be well discussed and selected together with the particular patient adapted to her/his individual needs. Importantly, a stepwise approach with initial conservative and later minimal invasive to invasive treatment is strongly recommended to minimize the risk of therapy-associated morbidity [37,38,39].

Current treatment options can be divided into three different categories: conservative, pharmacological and surgical [37,38]. The conservative options include pad usage, catheters or urinals, weight loss, smoking cessation, reducing unnecessary medication, behavioral therapy (i.e., bladder training, fluid/dietary modification, reducing caffeine intake) and physiotherapy as well as electrophysiological stimulation of the pelvic floor [11,19]. The key problems of pelvic-floor muscle training are lack of motivation, missing continuity and inconsistency in execution, all negatively affecting the training benefits of patients. An alternative to conservative treatment forms are the usage of vaginal pessaries or the insertion of urethral plugs.

From the Food and Drug Administration (FDA), there are no approved pharmacological treatments for SUI. Anticholinergic agents and/or beta-3 receptor agonists are most often used for clinical pictures of mixed urinary incontinence (SUI combined with overactive bladder). Additionally, the use of selective serotonin noradrenaline inhibitors (SSNRI), estrogens and desmopressin may be supportive. The available surgical options are manifold: paraurethral injection therapy (“bulking agents”), tension free vaginal tapes (TVTs), pubovaginal or mid-urethral urethral slings (retropubic vs. transobturatorial vs. autologous fascia), Burch colposuspension (open or laparoscopic) and artificial urinary sphincter and compression systems [11,40,41,42,43]. These interventions render a significant amelioration of the clinical symptoms and enable the recovery of continence with around 80% cure rates [44]. However, these options offer short-term relief only and they tend to cause complications in the short- and long-term perspective such as bladder or vaginal perforation, voiding or storage dysfunction (urinary retention or new onset urge UI), chronic pelvic pain, urethral and vaginal erosions or material infections [45]. In addition, around 10% of patients undergoing surgery for SUI will have worsening symptomatology due to new onset of detrusor overactivity (DOA) causing urgency incontinence [45,46]. This may lead to further operations or the need for explanation of the foreign materials.

## 3. The Regenerative Approach

To date, a definitive correction of the underlying etiology of SUI has not been accomplished. Therefore, the search for alternative solutions and regenerative treatment options are of paramount importance. TE is a field of regenerative medicine and includes the principles of cell transplantation, material science and bioengineering for the development of biological substitutes that may be used as autologous transplants to restore or maintain organ function. Precursor cells are isolated from living human tissue biopsies and thereafter multiplied in vitro. Later, these explanted cells are mixed with or seeded onto biodegradable polymers, and are replanted to repair or replace the injured or diseased tissue. The overall goal is the integration of the bioengineered cells into the surrounding tissue to rebuild a functional organ. Cell-based therapies are proposed as a method to achieve restoration of numerous tissues and organs. A huge advantage of autologous TE approaches over conventional organ transplantation is the fact that immunosuppression can be omitted, because the patient’s own cells are not rejected by the recipient’s immune system. Furthermore, patients are not dependent on the limited availability of transplantable organs. However, this approach is technically demanding, expensive and needs a broad combination of knowledge from different basic and clinical research fields.

**Figure 1:**
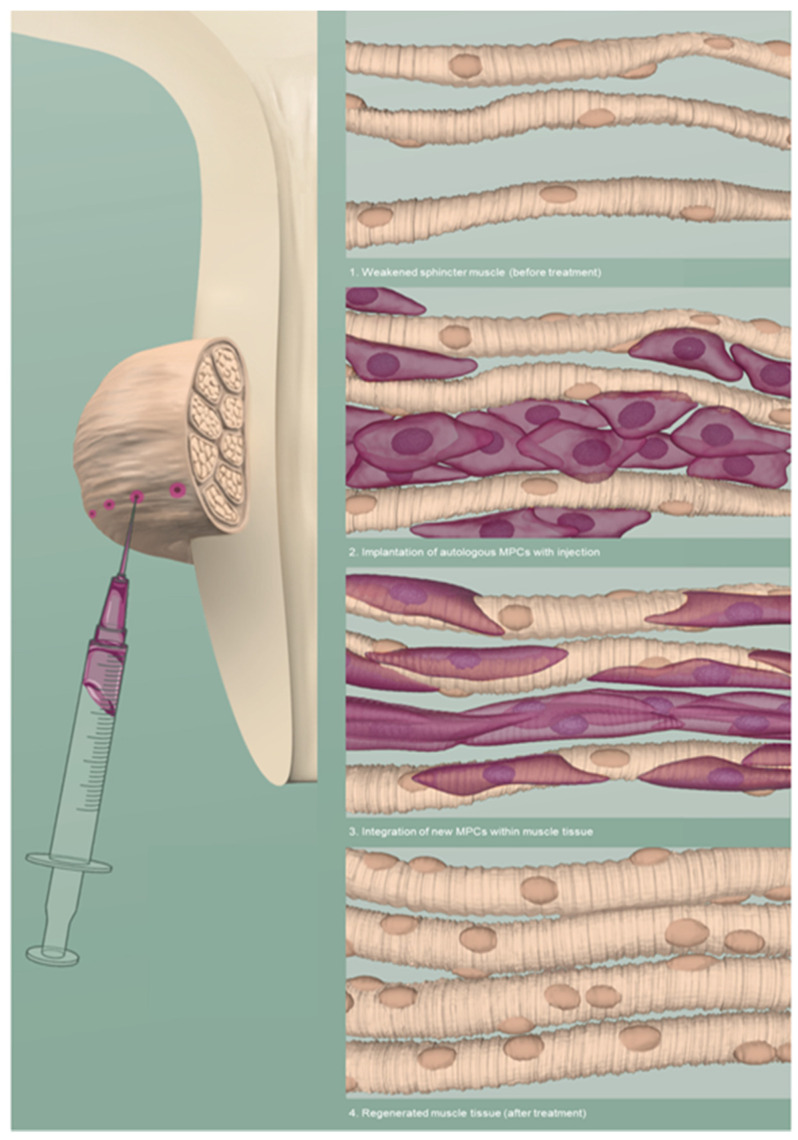
Schematic visualisation of an intrasphincteric injection of muscle precursor cells (MPCs) to regenerate the muscle tissue of the EUS. Adapted from © Jasmin Hegetschweiler and Nina Schwarz.

A particular option is the implantation of autologous muscle stem cells into the sphincter area to strengthen and restore EUS function [47]. Figure 1. The implanted stem cells would then increase MUCP to resist higher intra-abdominal strain [17]. Nevertheless, the process of cell isolation, expansion, harvesting and implantation is a complex, expensive and a critical endeavor, which is dependent on the effects of culture on cell biology at the molecular level.

Embryonal, umbilical cord and adult stem cells are most commonly used for such a treatment. Due to immunological, oncological and ethical considerations, research has focused on the latter for quite some time. Adult stem cells are mainly isolated from either adipose tissue, bone marrow or skeletal muscle [48]. Recent advances in cell-based therapies have provided a variety of opportunities to seek alternative solutions in restoring damaged sphincter function in patients with UI [49]. Functional restoration of the damaged EUS using the patient’s own cells would be an ideal treatment option that could reverse the underlying pathologic condition. The application of such treatments belongs to the category of advanced therapy medicinal products (ATMP), which underlie strict control mechanisms and various regulations from ethical, pharmacological and medical supervisory authorities. The stringent supervision serves as quality control and assures the transfer of relevant information within the involved study teams and between good clinical (GCP) and manufacturing practice (GMP). This is critical for translating a promising achievement from the laboratory bench to the patient’s bedside, and later into a specific treatment of clinical daily routine [50]. Many studies have explored the preclinical and clinical application of stem cells isolated from various origins that have promising results for the treatment of SUI [51,52,53,54,55,56,57,58,59,60,61,62,63,64,65] and were summoned up as part of the several reviews articles. Hart and colleagues summarized the mainly preclinical published literature on cell therapy models for the regeneration of smooth and striated urethral sphincter muscles, but also the regeneration of nerves and neuromuscular synapses [47]. Earlier, Lin et al. analyzed the literature in a review on different cell injection routes (transurethral, periurethral and intravenous), cell labeling, functional and histological assessment from mainly animal models of preclinical trials [66]. In 2016, Williams et al., mainly focusing on animal models, summarized the current state of knowledge on cell therapy for SUI introducing regenerative pharmacology as an adjunct or replacement [67]. A Chinese and American collaboration wrote a review with new aspects on the preclinical usage of tissue-engineered suburethral slings, that combine mechanical and regenerative aspects of stem cell therapy in SUI [48]. They also pointed out nicely the six biggest challenges to success in stem cell treatment. These are optimal dose and delivery route, proliferation and differentiation, distribution, abnormal differentiation or neoplastic transformation, nerve regeneration and adequate animal models to mirror chronic condition. Lastly, Vinarov and colleagues published a thorough literature review, putting together all the available evidence on preclinical and clinical studies using different stem cell types [68]. Nevertheless, the risk of aberrant cell populations is always present, which may lead to non-functional scar tissue (i.e., fibroblasts) or even tumor growth (i.e., teratoma). Hence, the main challenges are to avoid deviations of precursor cell lines and to lead them into the right branch of differentiation [50]. Further, the microenvironments of the tissue surroundings change significantly and paracrine effects such as those exerted by chemokines, secretomes, cytokines, growth and angiogenic factors become important determinants of cell fate [69].

### 3.1. Muscle Progenitor Cells (MPCs)

Regeneration of skeletal muscles is a highly orchestrated process that involves tight regulation of multiple cellular and molecular responses. Satellite cells can be found in the sublaminar layer of every skeletal muscle and are normally in a resting state. Upon certain stimuli (i.e., trauma or strain), they re-enter the cell cycle and differentiate into muscle progenitor cells (MPCs). The majority of MPCs are committed to the myogenic lineage and are therefore most suitable for muscle engineering. Extensive studies have examined this process in detail and have shown that MPCs are an ideal cellular model to study muscle tissue regeneration in the preclinical and clinical setting (see following paragraphs). Interestingly, MPC-based therapies have been proposed not only as a novel treatment option for SUI, but also in various other muscle diseases (genetic and acquired muscle disorders) [70,71].

#### 3.1.1. Preclinical Data

The use of injectable cultured MPCs for the treatment of SUI was analyzed in various animal studies, such as rodent, porcine, canine and primate models. Chancellor et al. introduced the general strategy of using adult stem cells for the treatment of UI in the early millennial years. These researchers injected muscle-derived stem cells (MDSCs) into rats and were able to show that the cells persisted longer in situ than bovine collagen as comparison [72,73]. Further, the injected MDSCs led to the formation of myotubes and myofibers in the histological analysis. Even more important was that the cells were not rejected, providing support that the injection of autologous cells was feasible and safe in such a model. Another early study demonstrated that MPCs harvested from limb skeletal muscles in mice may enhance the regeneration process of striated urinary sphincter muscle after 1 month [74]. A further study reported that the periurethral injection of allogenic MDSCs improved the leak point pressure (LPP) in sciatic nerve-transected female rats compared to sham treatment [75]. Highly promising results in dogs indicated that autologous MPCs were able to restore otherwise irreversibly damaged urinary sphincter function with up to 80% of the initial closure pressure values in urodynamic analysis [28]. The injected cells were histomorphologically analyzed after 6 months and were able to survive and form mature, functional tissue within the damaged region. The long-term effect on urinary sphincter deficiency after autologous MPC injection was also examined in a nonhuman primate model with a previously transected pudendal innervation [76]. The promising results showed sustained structural and functional restoration of the maximal urethral pressure after 12 months. However, multiple factors (such as age, stress and body weight) may negatively affect the efficacy of MPC treatment in chronic urinary sphincter deficiency [77]. A recent study from Zhou and colleagues was able to demonstrate the presence and location of tissue-resident progenitor cells in the EUS through label-retaining cells at 1, 2, 3, 4 and 8 weeks after intraperitoneal injection of 5-ethynyl-2-deoxyuridine [78]. The thickness of the striated muscle layer in the EUS developed faster than the smooth muscle layer after injection, whereas stem cells were located densely adherent to muscle fibers directly under the basal membrane of laminin. Overall, these animal studies indicate the feasibility of using autologous cells of muscle origin for functional restoration of urinary sphincter muscle in animal models with sphincter deficiency.

#### 3.1.2. Clinical Data

The translation of preclinical studies and their results to clinical application is highly demanding. Unfortunately, the treatment of urinary incontinence with MDSCs has fallen into disrepute in 2008 due to ethical concerns about research work, in which excellent short time results were presented [79,80]. The researchers added up to 50% of pure fibroblasts to the cell injections and used the same collagen concentration as chosen for conventional bulking interventions, a strategy known to show acceptable short-term results only. Their work was retracted due to irregularities in conduct of work, documentation of source data and obtainment of patient consent. Nonetheless, the same group continued their research with convincing results in female as well as male patients with SUI, followed-up until 1 year after the intervention [61,81,82].

Nevertheless, the general approach of using adult cells for the treatment of SUI was further pursued by Sèbe et al., who reported results from 12 female patients who received intrasphincteric injections of autologous MPCs isolated from a biopsy of deltoid muscle [83]. Ten out of 12 patients were either dry or improved on pad tests, while QoL improved in half of the patients after 1 year of follow-up. In 2012 and 2013, Blaganje and Lukanovic published two articles of ultrasound-guided myoblast injections into the EUS followed by electrical stimulation (to enhance cell integration) as 2-step treatment in SUI of 38 female patients [84,85]. At follow-up 6 months after the intervention, the results showed a well-tolerated procedure, no reported adverse effects and that more than 75% of patients were either cured or had an improved SUI. Gerullis et al. investigated the safety, efficacy and effect of transurethral injection of autologous MDSCs in the damaged urethral sphincter of 222 male patients with postprostatectomy incontinence [86]. The therapy was effective in only half of the patients, where the treatment led to either continence (12%) or improvement (42%) of clinical symptoms. Chancellor and his group published a clinical trial in 2013, where they injected 38 women with low or high doses of autologous MDSCs from biopsies of the quadriceps muscle [87]. Patients were able to choose a second injection of the same dose after 3 months and the follow-up ended 12 months after the last treatment. No serious adverse events or major complications were reported and the majority of patients experienced a 50% or greater reduction in pad weight (89% vs. 62%) and in reported stress leaks (78% vs. 53%), favoring the group with the higher dose of injected cells. In 2014, Chancellor et al. reported a study using pooled data from 80 female patients with different doses of autologous MDSCs injected (range: 10–200 million cells) [88]. The higher dose groups also tended to have a more favorable effect on continence, measured using pad weight, stress leaks and questionnaires. Yiou et al. tried to avoid the complex and costly process of stem cell isolation and expansion [89]. Therefore, the injected freshly isolated myofibers from the gracilis muscle carrying MPCs in five males and five females with ISD. Continence improved during the 12 months follow-up in four out of five women and their MUCP increased twofold. In men, the effect on continence was moderate, while MUCP recordings showed similar improvement. Gras et al. chose a similar approach with the implantation of freshly harvested and minced skeletal muscle [90]. In a cohort of 35 women with uncomplicated and complicated SUI, significant reductions in the mean number of leakages and amelioration of patient reported outcomes (PROs) through scores from questionnaires (ICIQ-SF = International Consultation on Incontinence Questionnaire–Short Form) were witnessed. Stangel-Wojcikiewicz et al. presented data on functional results with complete or partial improvement in 75% and a significantly improved QoL after two and four years of follow-up in 16 female patients treated for SUI with MPCs [91,92]. The longest follow-up of 36 months after treatment with injections of autologous MDSCs was presented by Sharifiaghdas et al. [93]. Seven out of 10 female patients with SUI due to trauma were either cured or experienced improved results on MUCP measured by urodynamics or in cough stress and 1 h pad tests. In 2018, Jankowski and colleagues performed a double-blind, randomized and placebo-controlled trial evaluating the safety and efficacy of autologous MDSCs and placebo in 50 female patients with SUI and 93 controls [94]. The enrollment of patients was halted at 60% of planned subjects due to an unexpectedly high placebo rate (90%). They concluded that primary efficacy endpoints need to be clinically meaningful and selection criteria well defined to lower placebo response rate and to reveal a possible treatment effect. A summary of the clinical data is presented as Supplementary Table S1.

### 3.2. Routes of Application

The route and precision of an injection therapy in SUI is of veritable importance to achieve an accurate effect of a regenerative or other minimal-invasive therapy form. Therefore, not only for the regenerative approach using MPCs, but also for bulking agents a precise injection into EUS is an indispensable premise. Several approaches using needle injection were described in the literature: (a) transurethral by either endoscopic or ultrasonographic guidance, (b) periurethral and (c) transperineal. Just recently, the feasibility and accuracy of a new transurethral injection technique for the purpose of MPC implantation was published [95]. Only a few studies have compared the outcomes of different injection routes among each other [96,97,98]. Studies including urethral injection therapies for the treatment of female SUI were analyzed in a recent Cochrane review, demonstrating no evidence for a clear superiority regarding a particular application form [99]. A novel technique described by Jaeger et al. used a needle-free method with waterjet technology to deliver mesenchymal stromal cells (MSCs) into the EUS [56]. The homogenous distribution of stem cells within the urinary sphincter through a “spray effect” may advance to the predominant method of injection in the future. A recently published manuscript by the same group has demonstrated superiority of the waterjet technology over endoscopic needle injections regarding on-site precision [100]. Additionally, the implantation of porcine adipose tissue-derived stromal cells (pADSCs) was faster and more homogenous with waterjet technology and showed morphologically intact and viable cells after histology of cryosections of the EUS.

### 3.3. Neuromuscular Electromagnetic Stimulation (NMES)

Implanted MPCs can be treated with external neuromuscular electromagnetic stimulation (NMES) to enhance cell proliferation and the differentiation into the myogenic lineage to ameliorate functionality through optimal embedding into the surrounding tissue. NMES has the ability to induce muscle contraction and has been proposed as a therapeutic modality for skeletal muscle diseases [101]. Magnetic stimulation also supports nerve and muscle regeneration by activating muscle-nerve cross-talk and inducing the maturation of neuro-muscular junctions in mice [102]. Further, NMES improves muscle regeneration by minimizing the presence of inflammatory infiltrate and the formation of scars after trauma [102]. It inhibits posttraumatic atrophy, induces hypertrophy and increases the metabolism and turnover of the muscle.

A therapeutic chair, inducing pulsed magnetic stimulation for the exercise of pelvic floor muscles in 120 females with SUI, has been shown to significantly improve ICIQ-scores in up to 75% of participants after 16-32 sessions and one year of follow-up [103]. A Japanese research team investigated the effect of magnetic stimulation on SUI in female patients being refractory to pelvic floor physiotherapy [104]. Patients were randomized 2:1 to either NMES or sham treatment: 30 individuals completed the trial with 24h pad tests, ICIQ-scores and QoL questionnaires that were significantly better compared to before NMES treatment as well as compared to the sham treatment group. An observational study from Vadala et al. reproduced similar findings in 10 female and 10 male patients suffering from SUI [105], where MUCP and urethral functional length increased significantly compared to baseline values.

### 3.4. Imaging Technologies for MPC Tracking

The fate of MPCs after implantation demands to be further investigated with non- or minimal-invasive methods. Therefore, novel imaging technologies are needed. The possibility of using positron emission tomography (PET) in CT imaging to non-invasively monitor implanted MPCs, that have been genetically modified to express dopamine 2 receptors (D2R), has been investigated [106]. 18F-Fallypride PET radioligands were successfully used to visualize the MPCs by fluorescent microscopy and confirmed sustained survival of the transplanted cells at different time-points with formulation of muscle tissue at the site of injection. Another approach utilized superparamagnetic iron oxide (SPIO) nanoparticles for the tracking of MPCs using magnetic resonance imaging and X-ray micro-computed tomography (µCT) in rat hearts [107]. Histological and imaging analysis revealed that iron-oxide particles were confined to viable, skeletal muscle-derived cells in the implant at the expected location based on magnetic resonance imaging (MRI) and μCT. No evidence of phagocytosis of labelled cells by macrophages was documented after 1 year. Others have used unified fusion reporter genes, whose expression can be monitored with bioluminescence imaging, for the localization of viable MPCs following implantation into a mouse model [108]. An advantage of this approach is the possibility to merge multiple imaging modalities. Recently published research presented the use of polymer-entrapped perfluorocarbons to label human cells, which can be visualized with photoacoustic imaging, Fluorine-19 (19F) MRI, US or fluorescence [109]. These nanoparticles enable the measurement of pharmacokinetics and biodistribution or the tracking of injected precursor cells [110,111,112]. However, all these techniques require the preparation of stem cells with radioligands, SPIOs, nanoparticles or antibodies for bioluminescence imaging and may even need imaging modalities using emission of radiation. A promising, non-invasive and non-radiogenic imaging method for the evaluation and the functional assessment of (pelvic) muscle structures are MR diffusion tensor imaging (DTI) [113,114,115,116,117] or magnetization transfer (MT) for the analysis of muscle tissue formation [118,119]. DTI and MT are able to provide a quantitative and qualitative measurement of the muscular microstructure and its physiological processes as well as of the fiber formation after in vivo myogenic differentiation of human MPCs [120,121].

### 3.5. Chemokine Therapy

Regenerative Pharmacology is a field of Regenerative Medicine that seeks to use small molecules (or combination of molecules) to stimulate the body to self-heal in situ [122]. It is distinct from standard pharmacotherapy, which is often limited to the amelioration of symptoms. This approach has the potential of bypassing the lengthy, expensive and complicated cell isolation and implant pre-conditioning protocols of conventional regenerative medicine. It is also one of the few remaining unexplored approaches for regenerating the lower urinary tract. Current efforts are underway to identify the cell secretions and determine their individual or combined regenerative efficacy. Lavoie et al. and Deng et al. found that the sum of secretions from progenitor cells stimulate structural and functional regeneration of the urethra [123,124]. Williams et al. have published that local sphincter administration of CXCL12 (also known as stromal cell-derived factor 1), without cell therapy, restores short-term urinary sphincter structure in NHPs with chronic sphincter deficiency [125]. An effect related to the ability of CXCL12, but not cells, to mobilize bone marrow cells and restore vascularization and innervation [125]. A cautionary note for this approach is that molecules do not function in a vacuum and interact with other molecules. As such, a thorough knowledge of their mechanisms of action are required. For instance, studies using recombinant CXCL12 injections have shown positive effects on tissue regeneration [125,126]. However, there are published studies that seem to contradict these results and dispute the beneficial effects of CXCL12 on urological tissues [127,128]. Nevertheless, these studies focused on the secretion of physiologic CXCL12 that Ray et al. showed to form dimers in physiologic conditions. High concentrations, which more efficiently activate pathways may lead to pathology and malignancy and inhibit chemotaxis [129]. Veldkamp et al. also suggested low concentrations of monomeric CXCL12, which stimulates and is the active form for chemotaxis [130,131]. Monomeric CXCL12 is secreted by both mammalian cells and bacteria and is commercially available in recombinant, purified form and used for current treatment injections. Thus, how the molecule is tested, the dose of the molecule and its interaction with other pathways are critical for the translation to human use.

## 4. Discussion and Future Prospects

SUI is not a deadly disease compared to cancer or cardiovascular entities and therefore treatment never of imminent character. Nevertheless, it is with no doubt a chronic condition that is associated with low QoL, many potential sequelae and high cumulative healthcare costs. A solution for SUI with its ever-increasing patient numbers and a pathophysiologically complex etiology is yet to be found. Despite the immense progress towards a successful implementation of cell-mediated therapies to treat SUI, several key aspects still need further investigation and improvement.

It will be necessary to enhance the survival and metabolism of the injected cells, in order to improve the quality and functionality of the bioengineered tissues. The tracking of long-term viability and function of implanted cells is critical–finding a tool to precisely and non-invasively monitor cell fate would be a major breakthrough. Herewith, an accurate implantation is an indispensable premise for MPCs to differentiate into striated EUS, which is the fundament to a reasonable subjective and objective clinical outcome at a later time point. Promising future techniques include direct visual control through an endoscopic approach, additional to the ultrasound guidance. Further, the use of innovative technologies such as waterjet injections demonstrated a homogeneous distribution of stem cells in the periurethral target region of the EUS. Some published articles have shown an impressive precision and excellent repeatability of such procedures in preclinical animal studies [56,100]. This groundbreaking technique will most probably receive the permission to be applied as future method of cell implantation in regenerative clinical trials treating SUI.

Long-term results can be monitored with either urodynamic studies or non-invasive and non-radiogenic imaging modalities. The advantage of functional MR imaging is the combination of cell-tracking and the assessment of EUS function at the same time, whereas urodynamic studies provide a functional evaluation only. An interesting future alternative for non-invasive in vivo imaging of MPCs after injection would be tracking with the help of nanoparticles (polymer-entrapped perfluorocarbons) in 19F MRI or US. Apart from imaging, the direct effect of NMES remains unclear to date. This needs to be further elucidated in prospective research trials, where muscle stem cells are used to treat SUI, in order to analyze the treatment effect of NMES as in subjective and objective functional results.

On the regenerative therapy side, there is potential for alternative strategies in the field of regenerative pharmacology, which already showed promising early results and need to be further developed. Chemokine treatment seeks to use small molecules (or a combination of molecules) to stimulate the body to self-heal in situ [122]. Current efforts are underway to identify the cell secretions and to determine their individual or combined regenerative efficacy. Surely, the dose of the molecule and its interaction with other pathways are critical for the translation to human use. To ameliorate the effect of chemokines, the use of stable and biocompatible drug-delivery systems could be a promising modification. Cubosomes are an example of lipid-based nanoparticles for the protected delivery of any biomacromolecule to the target region in order to enhance the in vivo efficiency of the carried drug [132]. Since therapeutics tend to be susceptible to degradation by enzymes, cubosomes would improve bioavailability and cellular uptake of specific pharmacological therapies in a correct dosage. However, upon cellular uptake the efficient release at its target location is the first challenge to consider when designing nanocarriers. Another possible strategy to ameliorate the regenerative treatment outcome in patients with SUI could be the repeated injections of autologous MPCs in the same patient. If we manage to establish freezing-thawing cycles without a relevant loss of cell count and viability, patients will not need additional muscle biopsies and the dosage of the applied MPCs could be adjusted. In combination with the stimulation of injected adult muscle stem cells through the application of NMES and/or ongoing pelvic floor physiotherapy, the ideal result would be a long-lasting functional effect.

## 5. Conclusions

Despite the immense progress towards the successful implementation of cell-mediated therapies to treat SUI, several key aspects still need further investigation and improvement. It will be necessary to enhance the survival and metabolism of the injected cells, in order to improve the quality of the bioengineered tissues. The non-invasive tracking of long-term viability and functionality of implanted cells is critical–finding a tool to monitor cell fate would be a major breakthrough. Finally, alternative approaches (e.g., regenerative pharmacology) need to be further developed as stand-alone or combined treatment strategies for patients with SUI. Overall, there is a huge potential for stem cell therapies in combination with regenerative pharmacology for the treatment of patients with SUI in the near future.

## Data Availability

Data sharing not applicable.

## Supplementary Materials

Supplementary materials can be found at https://www.mdpi.com/article/10.3390/ijms22083981/s1. Table S1. Summary of clinical data for the regenerative treatment of patients with SUI (Section 3.1.2.).

## Abbreviations

19F	Fluorine-19
ATMP	advanced therapy medicinal product
BMI	body-mass index
CXCL12	C-X-C Motif Chemokine Ligand 12
D2R	dopamine 2 receptors
DOA	detrusor overactivity
DTI	diffusion tensor imaging
EUS	external urethral sphincter
FDA	Food and Drug Administration
GCP	good clinical practice
GMP	good manufacturing practice
ICIQ	International Consultation on Incontinence Questionnaire
ICIQ-SF	International Consultation on Incontinence Questionnaire–Short Form
ICS	International Continence Society
ISD	intrinsic sphincter deficiency
LPP	leak point pressure
MDSC	muscle-derived stem cell
MPC	muscle progenitor cell
MRI	magnetic resonance imaging
MSC	mesenchymal stromal cell
MT	magnetization transfer
MUCP	maximal urethral closure pressure
NMES	neuromuscular electromagnetic stimulation
pADSC	porcine adipose tissue-derived stromal cell
PET	positron emission tomography
PRO	patient reported outcome
QoL	quality of life
SPIO	superparamagnetic iron oxide
SSNRI	selective serotonin noradrenaline inhibitors
SUI	stress urinary incontinence
TE	tissue engineering
TVT	tension free vaginal tape
UI	urinary incontinence
VLPP	Valsalva leak point pressure
µCT	micro-computed tomography
